# Healthy and Chronic Kidney Disease (CKD) Dogs Have Differences in Serum Metabolomics and Renal Diet May Have Slowed Disease Progression

**DOI:** 10.3390/metabo11110782

**Published:** 2021-11-16

**Authors:** Marcio Antonio Brunetto, Bruna Ruberti, Doris Pereira Halfen, Douglas Segalla Caragelasco, Thiago Henrique Annibale Vendramini, Vivian Pedrinelli, Henrique Tobaro Macedo, Juliana Toloi Jeremias, Cristiana Fonseca Ferreira Pontieri, Fernanda Maria Marins Ocampos, Luis Alberto Colnago, Marcia Mery Kogika

**Affiliations:** 1 Pet Nutrology Research Center, Nutrition and Production Department, School of Veterinary Medicine and Animal Science, University of São Paulo, Jardim Elite, Pirassununga 13635-900, Brazil; thiago.vendramini@usp.br (T.H.A.V.); henrique.tobaro@hotmail.com (H.T.M.); 2 Veterinary Nutrology Service, Veterinary Teaching Hospital, School of Veterinary Medicine and Animal Science, University of São Paulo, Cidade Universitária, São Paulo 05508-270, Brazil; dorisph2@yahoo.com.br (D.P.H.); vivian.pedrinelli@usp.br (V.P.); 3 Small Animal Internal Medicine Service, Veterinary Teaching Hospital, School of Veterinary Medicine and Animal Science, University of São Paulo, Cidade Universitária, São Paulo 05508-270, Brazil; brunaruberti@usp.br (B.R.); mv.douglas@yahoo.com.br (D.S.C.); mmkogika@usp.br (M.M.K.); 4 Nutrition Development Center, Grandfood Industria e Comercio LTDA (Premier Pet^®^), Dourado, São Paulo 05508-270, Brazil; jjeremias@premierpet.com.br (J.T.J.); cristiana@premierpet.com.br (C.F.F.P.); 5 Embrapa Instrumentação, R. 15 de Novembro, 1452, Centro, São Carlos 13560-970, Brazil; fmmocampos@gmail.com (F.M.M.O.); luiz.colnago@embrapa.br (L.A.C.)

**Keywords:** renal dogs, uremic toxins, metabolic profile, nutrition, survival

## Abstract

Chronic kidney disease (CKD) is highly prevalent in dogs, and metabolomics investigation has been recently introduced for a better understanding of the role of diet in CKD. This study aimed to compare the serum metabolomic profile of healthy dogs (CG) and dogs with CKD (CKD-T0 and CKD-T6) to evaluate whether the diet would affect metabolites. Six dogs (5 females; 1 male; 7.47 ± 2.31 years old) with CKD stage 3 or 4 (IRIS) were included. CG consisted of 10 healthy female dogs (5.89 ± 2.57 years old) fed a maintenance diet. Serum metabolites were analyzed by ^1^H nuclear magnetic resonance (^1^H NMR) spectra. Principal component analysis (PCA) and partial least squares discriminant analysis (PLS-DA) were performed to assess differences in metabolomic profiles between groups and before (CKD-T0) and after renal diet (CKD-T6). Data analysis was performed on SIMCA-P software. Dogs with CKD showed an altered metabolic profile with increased urea, creatinine, creatine, citrate, and lipids. Lactate, branched-chain amino acids (BCAAs), and glutamine were decreased in the CKD group. However, after 6 months of diet, the metabolite profiles of CKD-T0 and CKD-T6 were similar. Metabolomics profile may be useful to evaluate and recognize metabolic dysfunction and progression of CKD, and the diet may have helped maintain and retard the progression of CKD.

## 1. Introduction

Chronic kidney disease (CKD) is highly prevalent in dogs and is considered the most common renal disease in senile patients, though it occurs in all ages. CKD is characterized by progressive loss of renal function and is associated with high mortality [1,2]. The main function of the kidneys is the secretion of cytokines and hormones, excretion of waste metabolites, and the homeostasis of the electrolytes [2]. Consecutive failure to excrete waste metabolites may lead to uremic syndrome [2,3]. This syndrome is commonly observed in late-stage CKD patients [4] and may modify the biochemical and physiological functions of the animal. In humans, decreased renal function has been associated with hospitalization [5], cognitive dysfunction [6], and poor quality of life [7], and it is considered a global health burden with a high economic cost to the health system [8].

One of the recommendations to manage CKD progression and improve survival rate is the use of therapeutical diets [1,9,10,11]. Renal diets are designed to control the intake of phosphorus, protein, sodium, B-vitamin, and soluble fiber and are enriched with ω-3 polyunsaturated fatty acids (PUFAs) and antioxidants [12]. These diets attenuate oxidative stress [13,14,15,16] and the inflammatory course of the disease [16,17] while providing all the other nutrients required by the animal. Conversely, maintenance diets provide complete and balanced nutrition for the maintenance of the animal [18].

One of the challenges of studying the effects of renal diet interventions is the ability to assess slight changes in kidney metabolism [19]. The metabolomic approach offers an opportunity to obtain a comprehensive molecular picture of diet treatment and has been applied in nutrition research [20,21]. Metabolomics investigation involves the identification and quantification of small molecules that are present in biological samples and reflect the exogenous source of variation, aiming to highlight changes in individual metabolism under different conditions [22,23]. There are several methods of assessing the metabolomic profile of a sample; however, for covering the entire spectrum of metabolites, multiple analytical platforms are needed [24]. ^1^H nuclear magnetic resonance (^1^H NMR) is a commonly used high-throughput methodology that provides rapid and detailed molecule structure information, is nondestructive and reproducible, and allows for assessing changes in metabolite profiles in response to interventions [25,26]. This methodology has been applied in nutrition-related research [21,27] and has allowed for the identification of markers of renal disease [28,29].

This study aims to compare the serum metabolomic profile between healthy and CKD dogs and to assess whether diet intervention affects serum metabolites.

## 2. Results

**Figure 1:**
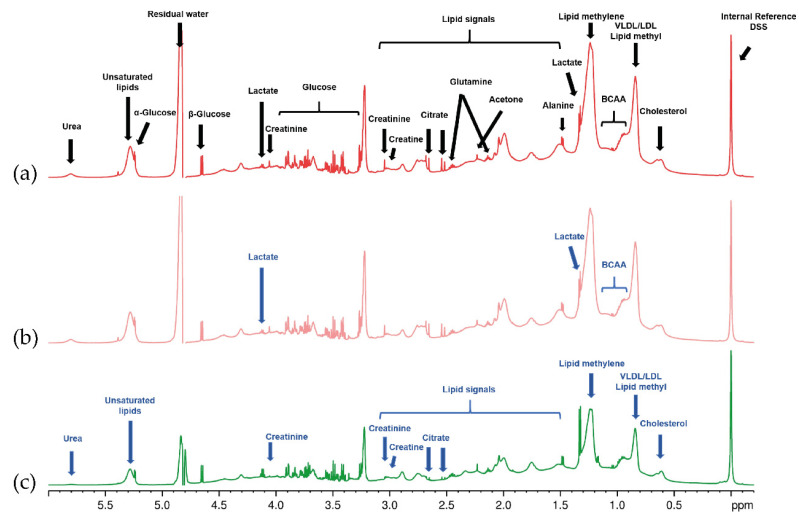
Expansion (−0.2–6.0 ppm) of 1D NOESY-presat ^1^H NMR spectra (600 MHz, 300 K) of the serum of the evaluated groups, indicating the main detected metabolites on the different samples. (**a**) Spectrum of the serum of CKD dogs after being fed a renal diet for 6 months (CKD-T6) highlighting all assignments of the metabolites’ NMR signals that have been identified; (**b**) spectrum of the serum of CKD dogs before being fed a renal diet for 6 months (CKD-T0), showing lactate and BCAAs in lower concentrations; (**c**) spectrum of the serum of healthy dogs (CG), highlighting the metabolites with a lower concentration than in both CKD groups (CKD-T0 and CKD-T6).

Dogs with CKD showed an altered metabolic profile, with increased levels of the following metabolites: urea, creatinine, creatine, citrate, and several lipids. Lactate, branched-chain amino acids (BCAAs), and glutamine concentrations decreased in the CKD dogs. However, after 6 months of renal diet (CKD-T6), we did not observe important metabolite differences in the CKD group before they were fed a renal diet (CKD-T0). Representative 1D NOESY-presat ^1^H NMR spectra with identified serum metabolites on the samples are shown in Figure 1.

Table 1 shows the assignments of the metabolites’ NMR signals, including several organic acids, lipoproteins, amino acids, and sugars. As the signal areas of the NMR spectra are directly correlated to the metabolite concentration (when acquired using the right acquisition parameters), this assay allows verifying which metabolites increased or decreased in concentration due to the disease process. A CPMG spectrum of the QC sample was acquired to filter large T2 molecules. It was used for the identification of metabolites (Figure S1). Likewise, 2D NMR correlation maps were important for correctly assigning and confirming the identified metabolites. HSQC, HMBC, and COSY 2D correlation maps are available in Figures S2–S4, respectively.

**Figure 2:**
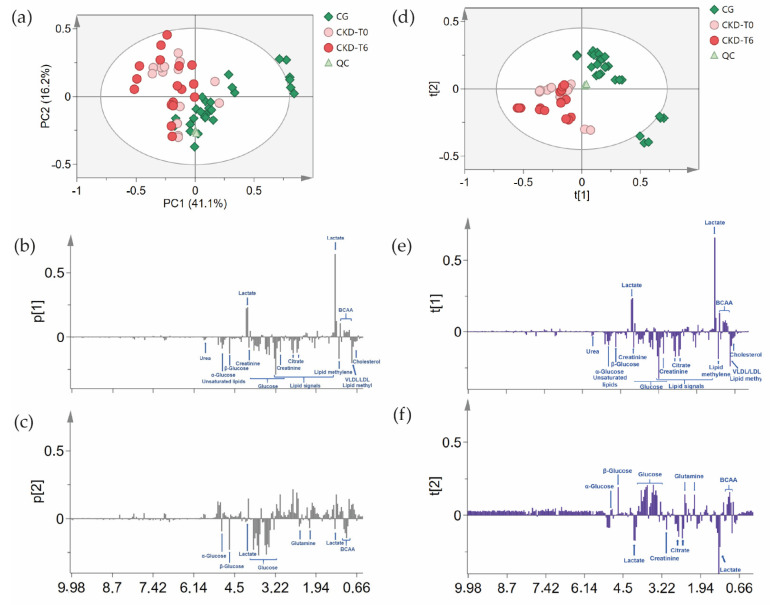
Principal component analysis (PCA) of ^1^H NMR data of dogs’ serum. (**a**) PCA score scatter plot of first principal component (PC1) versus second principal component (PC2); (**b**) PC1 loadings column plot of PCA, showing the main metabolites that influenced the separation; (**c**) PC2 loadings column plot of PCA, showing the main metabolites that influenced the separation; (**d**) PLS-DA score scatter plot of axis 1 versus axis 2; (**e**) loadings column plot of axis 1 of PLS-DA; (**f**) loadings column plot of axis 2 of PLS-DA, showing the main metabolites that influenced the separation. CG: control group of healthy animals; QC: quality control; CKD-T0: CKD dogs before being fed a renal diet; CKD-T6: CKD dogs after being fed a renal diet.

The untargeted unsupervised principal component analysis (PCA) (Figure 2) of 1D NOESY-presat spectra of dogs’ serum was used to evaluate the differences and similarities between the groups (CKD-T0 and CKD-T6), with the intent to verify whether the applied diet was capable of controlling the disease progression.

The designed model (Figure 2a–c) used seven principal components (PCs) to explain the variation of the data set and the variation of all the components explained by the model (R2X) was 84.7%. Figure 2a shows the score plot of principal components 1 and 2 (PC1 and PC2; PC1 and PC2 represented 57.3% of the data variance). The separation of the groups is given by PC1, as the CG had positive values and CKD dogs (CKD-T0 and CKD-T6) had negative values. Moreover, the PCA also demonstrates that the diet applied to the CKD dogs prevented the disease progression, as the CKD-T6 had a similar distribution to the CKD-T0 on the score scatter plot, clustering on negative PC1. The quality control sample (QC), which was reacquired during the process, shows the reproducibility of the NMR spectra acquisition process as they grouped. The CG had two animals located outside the 95% confidence level, although this may be explained by the biological variation, as the animals were client-owned and were not living under the same conditions.

As the PCA model recognized both sick groups (before and after diet) as being one, a supervised analysis was performed to try to discriminate the different groups and to identify the metabolites that significantly contributed to the classification. Thus, a partial least squares discriminant analysis (PLS-DA) model was created. The variance was explained by five components and the cumulative R^2^X was 72.8%. The R^2^Y and Q^2^ were higher than 0.5, indicating high-quality discrimination, with values of 0.6 and 0.5, respectively. Figure 2d–f show that the model was not able to completely distinguish the CKD-T0 and CKD-T6 groups, as they clustered together on negative axes 1 and 2 of the score scatter plot. The animals after the diet (CKD-T6) had slightly more negative values for both axes than the animals before the treatment. The loadings column plot for axis 1 was similar to the PCA, as the metabolites giving rise to the separation on this component turned out to be the same. On axis 2 of the loadings plot of PLS-DA, it was possible to verify that lactate and BCAAs were responsible for the CG clustering. Lactate may be the component with a higher concentration in the healthy group outside the 95% confidence level, as it is the only metabolite on positive axis 1 and negative axis 2. On the other hand, the CKD groups presented higher citrate and creatinine serum levels.

**Figure 3:**
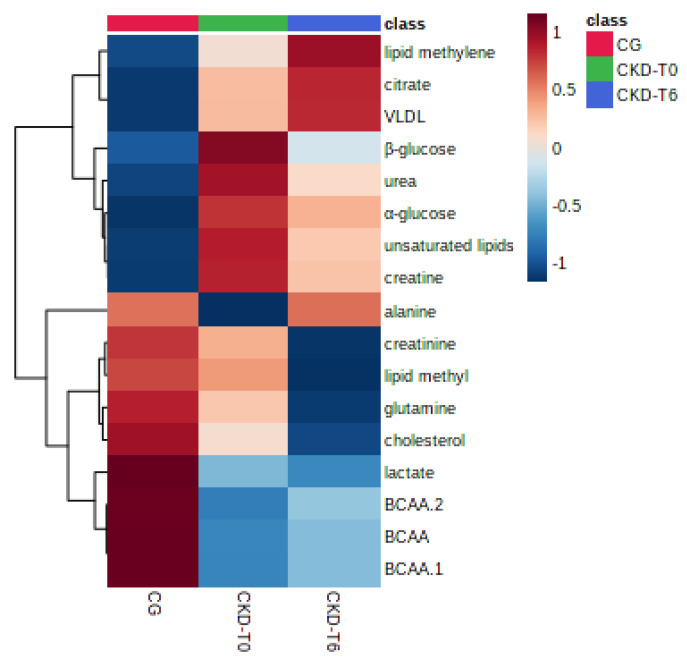
Hierarchical clustering heatmap of a targeted PLS-DA of ^1^H NMR data of dogs’ serum showing the main metabolites’ average levels for each group. CG: control group (healthy animals); CKD-T0: CKD dogs before being fed a renal diet; CKD-T6: CKD dogs after being fed a renal diet.

For a better appreciation of the metabolites that are present in each group, a targeted hierarchical clustering heatmap was developed (Figure 3). The heatmap confirmed that the group CKD-T0 has a similar metabolite profile to CKD-T6. However, differences between the sick groups were evidenced. CKD-T6 presented higher levels of saturated fatty acids, citrate, and VLDL, while sugars, unsaturated fatty acids, creatine, and urea values decreased after the diet. The levels of alanine increased, reestablishing the basal levels found in the control group. Creatinine, glutamine, and cholesterol presented even lower levels after the treatment. Levels of lactate and BCAAs were similar between both sick groups.

When we analyzed blood parameters, we noticed that there was no statistical difference in blood levels of urea (mean ± SE: CKD-T0 = 151.3 ± 43.3 vs. CKD-T6 = 118.7 ± 35.4 mg/dL; df = 5; t = 1.1; *p*-value = 0.32) and creatinine (CKD-T0 = 3.25 ± 0.5 vs. CKD-T6 = 3.5 ± 0.8 mg/dL; df = 5; t = −0.70; *p*-value = 0.51) between CKD-T0 and CKD-T6. Serum urea was greater in CKD-T0 compared to CG (CKD-T0 = 151.3 ± 43.3 vs. CG = 35.5 ± 5.4 mg/dL; df = 5; t = 2.65; *p*-value = 0.05). Similarly, serum creatinine was greater in CKD-T0 compared to CG (mean ± SE: CKD-T0 = 3.25 ± 0.5 vs. CG = 1.17 ± 0.05 mg/dL; df = 5; t = 4.11; *p*-value < 0.01).

## 3. Discussion

After a period of 6 months of renal diet intake, the metabolite profiles of CKD-T0 and CKD-T6 were similar. Disease progression is expected to be detectable after six months due to progressive loss of nephrons and thus reduced clearance of uremic toxins [1,2]. Our findings indicate that diet intervention may help to slow disease progression. In a recent work by our group, we found that six months of renal diet was able to control uremia, acid–base balance, blood pressure, antioxidant capacity, and production of inflammatory cytokines [16]. Similar results have also been described for humans [21].

We also compared the metabolomic profile of healthy and CKD dogs. We noticed marked differences in urea, creatinine, creatine, citrate, and a few lipids, all of them at higher concentrations in CKD dogs. Lactate, branched-chain amino acids (BCAAs), and glutamine, however, were observed at lower levels in CKD when compared to healthy dogs.

Urea is a small hydrosoluble molecule synthesized in the liver and is the end product of protein and nitrogen metabolism in mammals [30,31,32]. It is filtered by the glomerulus and then partially passively reabsorbed in the proximal and distal tubules [30,31]. When the urinary flow in the tubules is reduced due to the decrease in glomerular filtration rate (GFR), that passive reabsorption becomes increased [33] and then an increase in serum urea levels may occur in addition to the retention due to decreased GFR [30]. Urea is considered a renal function marker. Its level may be affected by dehydration, catabolic states, malnutrition, heart failure, use of glucocorticoids, hepatic urea synthesis, and gastrointestinal bleeding [30,34]. The amount of protein intake is intimately related to the amount of urea production; consequently, blood urea levels can be used to assess whether CKD patients in a renal diet are receiving adequate amounts of protein [35,36,37]. Hence, urea production levels can be used to estimate the accumulation degree of uremic toxins and be used as a guideline parameter for diet management in CKD patients [36,38].

Creatinine is a more specific biomarker used to estimate renal function, as it is associated with GFR [39,40] and exhibits repeatable signal intensities in the spectra. It is an amino acid from muscular catabolism, derived from the metabolism of phosphocreatine, freely filtered by the glomerulus, and secreted to a small degree by proximal tubular cells in dogs [40,41,42]. Total body creatinine production is directly related to the body muscle mass and can also be influenced by dietary intake [43], variation in tubular secretion, and extrarenal creatinine excretion [39,44,45].

Patients late stages of CKD usually have muscle wasting due to uremia, which activates the mechanism of cellular protein catabolism [46]. In the present study, although some animals lost weight, the dietary intake was able to maintain an ideal body condition score (BCS) [47] and prevented the loss of muscle mass score (MMS) in the majority of the animals. Patient survival has been associated with the maintenance of good BCS and MMS in CKD [48,49].

We also detected creatine as a retention solute, which is a nitrogenous organic acid derived from glycine, L-arginine, and S-adenosyl-L-methionine [50]. This metabolite is considered an essential contributor to cellular energy homeostasis [51]. In a reaction catalyzed by creatine phosphokinase (CPK), most of the creatine that is stored in the muscle is phosphorylated to creatine phosphate and nonenzymatically converted to creatinine, which is excreted by the kidneys [34].

In humans, in addition to endogenous synthesis, creatine is absorbed from the diet, especially from meat [52]. In dogs, Harris et al. [53] investigated many canine feedstuffs and found very low levels of creatine in processed feedstuff when compared to unprocessed sources. The heat processing of meat protein has a negative impact on creatine concentrations [53,54]. Hence, it is likely that the increased creatine levels found in our study were due to an inadequate renal clearance and not because of protein intake.

Another metabolite that showed accumulation in the serum was citrate. The mechanism supporting this result may be attributed either to an impairment of the Krebs cycle or to renal tubular acidosis, which typically appears as part of a generalized proximal tubule dysfunction, commonly observed in CKD patients according to Laing et al. [55]. Citrate is a molecule produced in the Krebs cycle and is transported by the kidney [56]. A study in humans revealed that decreased excretion of citrate is caused by tubulointerstitial lesions [57].

Our results showed that CKD dogs had increased levels of lipids. Lipidic disorders are common in human patients with kidney disease, especially glomerulonephritis [58]. Vaziri [59] demonstrated that during the progression of CKD in humans, it is common that the patients present hypercholesterolemia and elevated low-density lipoprotein (LDL) levels. In dogs, dyslipidemia has been reported in CKD patients [60]; the author found a decrease in high-density lipoprotein (HDL) and variable increases in LDL and very-low-density lipoprotein (VLDL). In fact, the combination of dysregulation of HDL and triglyceride-rich lipoprotein metabolism are some abnormalities that cause lipid dysregulation in CKD patients [59,60,61].

Decreased concentrations of lactate, a product of glycolysis, were found in CKD dogs. The kidney is a major organ for lactate removal in the body [62] even when there is a reduction in renal blood flow. Bellomo et al. [63] demonstrated that the kidney continues to remove lactate from the circulation even when 70% of its function is lost. Another study showed that lactate production is directly related to renal function; when kidney filtration was ceased, lactate production was inhibited [64].

Lower levels of BCAAs in CKD dogs were also observed. Abnormalities in BCAAs have been described in CKD human patients showing that low-protein diets could decrease these amino acids [65]. Metabolic acidosis can also stimulate the acceleration of BCAA catabolism. This condition can increase valine and leucine decarboxylation in muscle, providing lower BCAA plasma levels [66].

Another finding which may indicate that the renal diet treatment was insufficient to control metabolic acidosis was the lower glutamine level in CKD dogs. In the mitochondria of the proximal tubule cells, glutamine is converted to glutamate during metabolic acidosis. At the end of this reaction, glucose and two bicarbonate molecules are generated, and these mechanisms can help restore the systemic acid–base imbalance [67]. We did not measure the enzymes responsible for this transformation, which limits the conclusion of the occurrence of this hypothesis.

The limitations of the present study include reduced sample size and lack of a positive control group. Although enrolling a positive control group would be ideal to test diet efficacy, it would also mean a lower standard of care for the experimental animals, hence being unethical. We believe that future studies should increase sample size and follow more animals for a longer period while collecting samples at more time points. This would allow researchers to investigate when the diet begins to affect serum metabolites. Furthermore, future studies should evaluate the potential use of metabolomics for early identification of metabolic dysfunction in CKD and for follow-up and monitoring of the treatment.

## 4. Materials and Methods

### 4.1. Animals and Study Design

This study was conducted at the Veterinary Teaching Hospital of the School of Veterinary Medicine and Animal Science of the University of São Paulo (FMVZ/USP), São Paulo, SP, Brazil, and approved by the Ethics Committee of the Veterinary Medicine and Animal Science School of the University of São Paulo (FMVZ/USP), protocol number 3138/2013. The research was a prospective, 6-month longitudinal dietary trial utilizing a before (T0) and after (T6) design (Figure S2).

The first experimental group (CKD-T0) was composed of six dogs (1 male and 5 females) of various breeds (golden retriever, Labrador retriever, English bulldog, American pit bull terrier, and 2 mongrels), with a mean age of 7.47 ± 2.31 years, mean body weight of 13.25 ± 5.78 kg, mean BCS of 5.74 ± 0.80 [47], and mean MMS of 2.20 ± 0.50 [66]. These animals were diagnosed with CKD based on persistent azotemia over at least three months and were classified as stage 3 (n = 2) or 4 (n = 4) according to IRIS [68], and in routine urinalysis, these dogs did not show renal proteinuria. The dogs had a stable renal function, without symptoms such as anorexia or impairment of appetite, nausea/vomiting, or associated conditions, and had not consumed a renal diet previously. The first experimental group of dogs with chronic kidney disease started to eat a renal diet (CKD-T0) and after 6 months became the second experimental group (CKD-T6).

The third group was the control group (CG) and consisted of 10 healthy adult female dogs (mongrels), with a mean age of 5.89 ± 2.57 years, mean body weight of 14.96 ± 4.35 kg, mean BCS of 5.20 ± 0.42, and mean MMS of 2.50 ± 0.50. All dogs of mentioned groups underwent a complete physical examination, complete blood count, and biochemical serum profile (albumin, glucose, total protein, urea, creatinine, alkaline phosphatase, cholesterol, triglycerides, aspartate aminotransferase (AST), and alanine aminotransferase (ALT)).

### 4.2. Diet and Feeding Protocol

Nutritional contents of the diet were balanced and met all requirements for maintenance of adult dogs [18,69]; however, the diet had a baseline concentration of protein and phosphorus and the addition of ω-3 PUFAs and vitamin E, as described in Table 1.

Owners received the recommendation that no other food should be provided. There was a 4-day adaptation period to the new diet when prior food and the experimental diet were mixed. The amount of food to be fed daily was calculated using the following equation: 95 kcal × (body weight)^0.75^ [18]. The result was divided by the metabolizable energy of the diet to calculate daily food intake.

The control group received a maintenance diet (Golden Formula—Adult Dogs/Chicken and Rice, Grandfood Industry and Commerce Ltd., Dourado, Brazil (Premier Pet)). This group was evaluated only as a reference group consuming maintenance food. Chemical composition per kg of natural matter and per 1000 kcal and ingredients are presented in Table 2.

The maintenance energy requirement (MER) of each animal was calculated by the equation: MER = 95 × (body weight)^0.75^ = kcal/day [18,69].

### 4.3. Sample Collection and Preparation

All blood samples were collected in the morning period using a needle and syringe by venipuncture and transferred into red top vacutainer tubes containing clot activator. For ^1^H NMR analysis, serum was separated by centrifugation (1600× *g* for 15 min) within 30 min of collection and frozen immediately at −80 °C. A QC sample was prepared by mixing 10 µL of every serum sample. Serum aliquots (200µL) were added to 400 µL of phosphate buffer (pH 7.4) in D_2_O, centrifuged at 12,000× *g* for 5 min, and transferred into 5 mm NMR tubes [70].

### 4.4. NMR Spectral Acquisition, Processing Parameters, and Identification of Serum Metabolites

All NMR experiments were performed at 300 K on a Bruker AVANCE III HD 600 NMR spectrometer operating at 14.1 T, observing ^1^H and ^13^C at 600.13 and 150.90 MHz, respectively. The spectrometer was equipped with a 5 mm multinuclear detection probe with a z-gradient. The QC sample was used to calibrate the 90° pulse length, to determine the offset of the water signal for the water suppression, and to periodically monitor acquisition. ^1^H NMR spectra (128 transients and 32 K data points) were acquired with ICON software for automation of acquisition parameters, using 1D NOESY-presat (*noesygppr1d*) and CPMG-presat (*cpmgpr1d*) pulse sequences on a spectral width of ~20ppm. For 1D NOESY-presat spectra acquisition, a mixing time of 100 ms and relaxation delay of 2 s and 4 dummy scans were used. CPMG-presat spectra were acquired with 16 dummy scans, a relaxation delay of 2 s, and 80 loops. One-bond (HSQC) and long-range (HMBC) ^1^H-^13^C NMR correlation experiments were optimized for average coupling constants ^1^J_(H,C)_ and ^LR^J_(H,C)_ of 140 and 8 Hz, respectively. Spectra were processed using TopSpin 3.5 software. All ^1^H and ^13^C NMR chemical shifts were observed in ppm related to DSS signal at 0.00 ppm as an internal reference, and an exponential line broadening of 0.3 Hz was applied. After Fourier transformation, spectra were manually phased (zero and first phase) and baselines were corrected. Metabolites were assigned based on the chemical shifts and signal multiplicities, using Chenomix software [70].

### 4.5. Statistical Analysis

The multivariate analysis of the ^1^H NMR spectra of the 22 serum samples was conducted in SIMCA-P software (version 14.1, Umetrics). Whenever the amount of serum was enough, sample preparation triplicates were performed, and the 3 QC sample spectra were included in the data set in order to verify reproducibility. For this purpose, simple rectangular buckets of 0.04 ppm were calculated using AMIX software using the special integration mode and scaling to total intensity. The analysis included the regions between 0.2 and 10 ppm, excluding the water residual signal (4.7–5.1 ppm). The principal component analysis (PCA, 64 spectra × 236 columns) and the partial least squares discriminant analysis (PLS-DA, 64 observations, 240 variables (X = 236, Y = 4) were performed using Pareto scaling preprocessing on the data set. The hierarchical clustering heatmap of a targeted PLS-DA was performed using the MetaboAnalyst 5.0 (http://www.metaboanalyst.ca/, accessed on 18 October 2021) online tool, in which data were normalized by median and scaled using Pareto preprocessing.

Differences in blood parameters (urea and creatinine) between CG and CKD-T0 were analyzed through two-sample unequal variance t-test. Comparison of blood parameters (urea and creatinine) between CKD-T0 and CKD-T6 was analyzed using paired t-test.

## 5. Conclusions

Healthy and CKD dogs had marked differences in serum metabolomics, indicating that this tool may be useful to evaluate and recognize the metabolic dysfunction along with the progression of the disease. CKD dogs after six months of diet intervention showed similar metabolomics when compared to the beginning of the treatment, suggesting that diet may have helped the maintenance and slowed the progression of CKD.

## Figures and Tables

**Table 1:** Diet composition ^a^ as fed and per 1000 kcal and ingredients * according to the manufacturer of the renal diet used in the study.

Nutrients		
	Per 100 g of Diet (as Fed)	Per 1000 kcal
Dry matter (g)	90.00	−
Protein (g)	14.50	35.60
Fat (g)	18.00	44.20
Ash (g)	5.50	13.50
Crude fiber (g)	3.50	8.60
Minimum calcium (g)	0.40	0.98
Maximum calcium (g)	0.90	2.21
Phosphorus (g/kg)	0.30	0.74
Potassium (g/kg)	0.60	1.47
Omega 6 (g)	2.00	4.91
Omega 3 (g)	0.52	1.27
EPA + DHA (g)	0.35	0.86
Food base excess (mEq)	11.30	27.75
Metabolizable energy (kcal/g)	4.072 ^b^	

^a^ Premier Nutrição Clínica Renal Cães—Grandfood Industry and Commerce Ltd., Dourado, Brazil (Premier Pet). ^b^ Metabolizable energy of the diet, previously calculated in a metabolism assay at the Nutritional Development Center—Grandfood Industry and Commerce Ltd., Dourado, Brazil (Premier Pet). * Ingredients: poultry meal, soy protein isolate, spray-dried egg, broken rice, ground whole corn, barley, beet pulp, poultry fat, stabilized animal fat, fish oil, hydrolyzed poultry, antioxidant BHA, potassium citrate, potassium chloride, dried brewer’s yeast, vitamin, and mineral premix.

**Table 2:** Diet composition ^a^ as fed and per 1000 kcal and ingredients * according to the manufacturer of the control diet used in the study.

Nutrients		
	Per 100 g of Diet (as Fed)	Per 1000 kcal
Dry matter (g)	90.00	−
Protein (g)	23.00	60.61
Fat (g)	12.00	31.62
Ash (g)	7.50	19.76
Crude fiber (g)	3.00	7.91
Minimum calcium (g)	0.80	2.11
Maximum calcium (g)	1.60	4.22
Phosphorus (g/kg)	0.70	1.84
Potassium (g/kg)	0.50	1.32
Omega 6 (g)	2.00	5.27
Omega 3 (g)	0.22	0.58
Metabolizable energy (kcal/g)	3.795 ^b^	

^a^ Golden Fórmula—Cães Adultos/Frango e Arroz—Grandfood Industry and Commerce Ltd., Dourado, Brazil (Premier Pet). ^b^ Metabolizable energy of the diet, previously calculated in a metabolism assay at the Nutritional Development Center—Grandfood Industry and Commerce Ltd., Dourado, Brazil (Premier Pet). * Ingredients: meat meal, poultry by-product meal, isolated pork protein, whole-grain cornmeal, broken rice, beet pulp, defatted rice bran, poultry fat, pork fat, flaxseed, pork and chicken hydrolysate, propionic acid, antioxidants BHA and BHT, potassium chloride, sodium chloride, dried brewer’s yeast, yeast cell wall, vitamin A, vitamin B12, vitamin C, vitamin D3, vitamin E, vitamin K3, folic acid, pantothenic acid, biotin, choline chloride, niacin, pyridoxine, riboflavin, thiamine, potassium iodide, selenium proteinate, copper f sulfate, iron f sulfate, manganese sulfate, zinc sulfate.

## Data Availability

The data presented in this study are available on request from the corresponding author.

## Supplementary Materials

The following are available online at https://www.mdpi.com/article/10.3390/metabo11110782/s1, Figure S1. Comparison between 1D NOESY-presat and 1D CPMG ^1^H NMR spectra (600 MHz, 300 K) of the QC sample, indicating the chemical shifts of the main detected metabolites of dogs’ serum, demonstrating the effect of the T2 filter. Figure S2. Periods, animals, and experimental procedures that were performed in the study. Figure S3. Expansion (6.0–0.2 ppm) of HSQC correlation map (^1^H and ^13^C at 600.13 and 150.90 MHz, respectively, 300 K) of the QC sample from dogs’ serum. Figure S4. Expansion (6.0–0.2 ppm) of HMBC correlation map (^1^H and ^13^C at 600.13 and 150.90 MHz, respectively, 300 K) of the QC sample from dogs’ serum. Figure S5. Expansion (6.0–0.2 ppm) of COSY correlation map (^1^H at 600.13 MHz, 300 K) of the QC sample from dogs’ serum.

## References

[1] Polzin D.J., Bartges J., Polzin D. (2011). Chronic Kidney Disease. Nephrology and Urology of Small Animals.

[2] Bartges J.W. (2012). Chronic Kidney Disease in Dogs and Cats. Vet. Clin. N. Am. Small Anim. Pract..

[3] Vanholder R., De Smet R., Hsu C., Vogeleere P., Ringoir S. (1994). Uremic toxicity: The middle molecule hypothesis revisited. Semin. Nephrol..

[4] Vanholder R., De Smet R. (1999). Pathophysiologic effects of uremic retention solutes. J. Am. Soc. Nephrol..

[5] Go A.S., Chertow G.M., Fan D., McCulloch C.E., Hsu C. (2004). Chronic Kidney Disease and the Risks of Death, Cardiovascular Events, and Hospitalization. N. Engl. J. Med..

[6] Etgen T., Chonchol M., Förstl H., Sander D. (2012). Chronic kidney disease and cognitive impairment: A systematic review and meta-analysis. Am. J. Nephrol..

[7] Perlman R.L., Finkelstein F.O., Liu L., Roys E., Kiser M., Eisele G., Burrows-Hudson S., Messana J.M., Levin N., Rajagopalan S. (2005). Quality of life in Chronic Kidney Disease (CKD): A cross-sectional analysis in the Renal Research Institute-CKD study. Am. J. Kidney Dis..

[8] Hill N.R., Fatoba S.T., Oke J.L., Hirst J.A., O’Callaghan C.A., Lasserson D.S., Hobbs F.D.R. (2016). Global Prevalence of Chronic Kidney Disease—A Systematic Review and Meta-Analysis. PLoS ONE.

[9] Polzin D., Osborne C., Hayden D., Stevens J. (1984). Influence of reduced protein diets on morbidity, mortality, and renal function in dogs with induced chronic renal failure. Am. J. Vet. Res..

[10] Jacob F., Polzin D.J., Osborne C.A., Allen T.A., Kirk C.A., Neaton J.D., Lekcharoensuk C., Swanson L.L. (2002). Clinical evaluation of dietary modification for treatment of spontaneous chronic renal failure in dogs. J. Am. Vet. Med. Assoc..

[11] Chew D.J., Dibartola S.P., Schenck P. (2011). Canine and Feline Nephrology and Urology.

[12] Polzin D.J. (2013). Evidence-based step-wise approach to managing chronic kidney disease in dogs and cats. J. Vet. Emerg. Crit. Care.

[13] Yu S., Gross K., Allen T. (2006). A renal food supplemented with vitamins E, C and beta-carotene reduces oxidative stress and improves kidney function in client-owned dogs with stages 2 or 3 kidney disease. J. Vet. Intern. Med..

[14] Yu S., Paetau-Robinson I. (2006). Dietary supplements of vitamins E and C and β-carotene reduce oxidative stress in cats with renal insufficiency. Vet. Res. Commun..

[15] Brown S.A. (2008). Oxidative Stress and Chronic Kidney Disease. Vet. Clin. N. Am. Small Anim. Pract..

[16] Halfen D.P., Caragelasco D.S., De Souza Nogueira J.P., Jeremias J.T., Pedrinelli V., Oba P.M., Ruberti B., Pontieri C.F.F., Kogika M.M., Brunetto M.A. (2019). Evaluation of electrolyte concentration and pro-inflammatory and oxidative status in dogs with advanced chronic kidney disease under dietary treatment. Toxins.

[17] Brown S.A., Brown C.A., Crowell W.A., Barsanti J.A., Allen T., Cowell C., Finco D.R. (1998). Beneficial effects of chronic administration of dietary ω-3 polyunsaturated fatty acids in dogs with renal insufficiency. J. Lab. Clin. Med..

[18] (2006). National Research Council (NRC) Nutrient Requirements of Dogs and Cats.

[19] Jones D.P., Park Y., Ziegler T.R. (2012). Nutritional Metabolomics: Progress in Addressing Complexity in Diet and Health. Annu. Rev. Nutr..

[20] Deng P., Jones J.C., Swanson K.S. (2014). Effects of dietary macronutrient composition on the fasted plasma metabolome of healthy adult cats. Metabolomics.

[21] Rebholz C.M., Zheng Z., Grams M.E., Appel L.J., Sarnak M.J., Inker L.A., Levey A.S., Coresh J. (2019). Serum metabolites associated with dietary protein intake: Results from the Modification of Diet in Renal Disease (MDRD) randomized clinical trial. Am. J. Clin. Nutr..

[22] Fiehn O. (2002). Metabolomics—The link between genotypes and phenotypes. Plant Mol. Biol..

[23] German J.B., Hammockc B.D., Steven M.W. (2005). Metabolomics: Building on a century of biochemistry to guide human health. Metabolomics.

[24] Xiao J.F., Zhou B., Ressom H.W. (2012). Metabolite identification and quantitation in LC-MS/MS-based metabolomics. TrAC Trends Anal. Chem..

[25] Coen M., Holmes E., Lindon J.C., Nicholson J.K. (2008). NMR-based metabolic profiling and metabonomic approaches to problems in molecular toxicology. Chem. Res. Toxicol..

[26] Qi S., Ouyang X., Wang L., Peng W., Wen J., Dai Y. (2012). A Pilot Metabolic Profiling Study in Serum of Patients with Chronic Kidney Disease Based on 1H-NMR-Spectroscopy. Clin. Transl. Sci..

[27] Savorani F., Rasmussen M.A., Mikkelsen M.S., Engelsen S.B. (2013). A primer to nutritional metabolomics by NMR spectroscopy and chemometrics. Food Res. Int..

[28] Shah V.O., Townsend R.R., Feldman H.I., Pappan K.L., Kensicki E., Vander Jagt D.L. (2013). Plasma metabolomic profiles in different stages of CKD. Clin. J. Am. Soc. Nephrol..

[29] Goraya N., Wesson D.E. (2015). Dietary interventions to improve outcomes in chronic kidney disease. Curr. Opin. Nephrol. Hypertens..

[30] Dossetor J.B. (1966). The Relative Significance of Blood Urea Nitrogen and Serum Creatinine Concentrations in Azotemia. Ann. Intern. Med..

[31] Kobayashi T., Patel V.B., Preedy V.R. (2015). Metabolomics and Stages of Chronic Kidney Disease. Biomarkers in Kidney Disease.

[32] Braun J.-P., Lefebvre H.P., Kaneko J.J., Harvey J.W., Bruss M.L. (2008). Kidney Function and Damage. Clinical Biochemistry of Domestic Animals.

[33] Park R., Rabinowitz L. (1969). Effect of Reduced Glomerular Filtration Rate on the Fractional Excretion of Urea in the Dog. Exp. Biol. Med..

[34] Hosten A.O., Walker H., Hall W., Hurst J. (1990). BUN and Creatinine. Clinical Methods: The History, Physical, and Laboratory.

[35] Maroni B.J., Steinman T.I., Mitch W.E. (1985). A method for estimating nitrogen intake of patients with chronic renal failure. Kidney Int..

[36] Elliott J., Rawlings J.M., Markwell P.J., Barber P.J. (2000). Survival of cats with naturally occurring chronic renal failure: Effect of dietary management. J. Small Anim. Pract..

[37] Franch H.A., Mitch W.E. (2009). Navigating Between the Scylla and Charybdis of Prescribing Dietary Protein for Chronic Kidney Diseases. Annu. Rev. Nutr..

[38] Weiner I.D., Mitch W.E., Sands J.M. (2015). Urea and Ammonia Metabolism and the Control of Renal Nitrogen Excretion. Clin. J. Am. Soc. Nephrol..

[39] Wyss M., Kaddurah-Daouk R. (2000). Creatine and creatinine metabolism. Physiol. Rev..

[40] Stevens L.A., Coresh J., Greene T., Levey A.S. (2006). Assessing kidney function—Measured and estimated glomerular filtration rate. N. Engl. J. Med..

[41] O’Connell J.M.B., Romeo J.A., Mudge G.H. (1962). Renal tubular secretion of creatinine in the dog. Am. J. Physiol. Content.

[42] Stevens L.A., Levey A.S. (2005). Measurement of kidney function. Med. Clin. N. Am..

[43] Preiss D.J., Godber I.M., Lamb E.J., Dalton R.N., Gunn I.R. (2007). The influence of a cooked-meat meal on estimated glomerular filtration rate. Ann. Clin. Biochem..

[44] Schutte J.E., Longhurst J.C., Gaffney F.A., Bastian B.C., Blomqvist C.G. (1981). Total plasma creatinine: An accurate measure of total striated muscle mass. J. Appl. Physiol. Respir. Environ. Exerc. Physiol..

[45] Levey A.S. (1990). Measurement of renal function in chronic renal disease. Kidney Int..

[46] Wang X.H., Mitch W.E. (2014). Mechanisms of muscle wasting in chronic kidney disease. Nat. Rev. Nephrol..

[47] Laflamme D. (1997). Development and Validation of a Body Condition Score System for Dogs. Canine Pract..

[48] Parker V., Freeman L. (2011). Association between body condition and survival in dogs with acquired chronic kidney disease. J. Vet. Intern. Med..

[49] Rudinsky A.J., Harjes L.M., Byron J., Chew D.J., Toribio R.E., Langston C., Parker V.J. (2018). Factors associated with survival in dogs with chronic kidney disease. J. Vet. Intern. Med..

[50] Walker J.B. (1979). Creatine: Biosynthesis, Regulation, and Function. Adv. Enzymol. Relat. Areas Mol. Biol..

[51] Brosnan J.T., Brosnan M.E. (2007). Creatine: Endogenous Metabolite, Dietary, and Therapeutic Supplement. Annu. Rev. Nutr..

[52] Balsom P.D., Söderlund K., Ekblom B. (1994). Creatine in Humans with Special Reference to Creatine Supplementation. Sports Med..

[53] Harris R.C., Lowe J.A., Warnes K., Orme C.E. (1997). The concentration of creatine in meat, offal and commercial dog food. Res. Vet. Sci..

[54] Dobenecker B., Braun U. (2015). Creatine and creatinine contents in different diet types for dogs—Effects of source and processing. J. Anim. Physiol. Anim. Nutr..

[55] Laing C.M., Toye A.M., Capasso G., Unwin R.J. (2005). Renal tubular acidosis: Developments in our understanding of the molecular basis. Int. J. Biochem. Cell Biol..

[56] Choi J.Y., Yoon Y.J., Choi H.J., Park S.H., Kim C.D., Kim I.S., Kwon T.H., Do J.Y., Kim S.H., Ryu D.H. (2011). Dialysis modality-dependent changes in serum metabolites: Accumulation of inosine and hypoxanthine in patients on haemodialysis. Nephrol. Dial. Transplant..

[57] Psihogios N.G., Kalaitzidis R.G., Dimou S., Seferiadis K.I., Siamopoulos K.C., Bairaktari E.T. (2007). Evaluation of tubulointerstitial lesions’ severity in patients with glomerulonephritides: An NMR-based metabonomic study. J. Proteome Res..

[58] Jia L., Wang C., Zhao S., Lu X., Xu G. (2007). Metabolomic identification of potential phospholipid biomarkers for chronic glomerulonephritis by using high performance liquid chromatography-mass spectrometry. J. Chromatogr. B.

[59] Vaziri N.D. (2006). Dyslipidemia of chronic renal failure: The nature, mechanisms, and potential consequences. Am. J. Physiol. Ren. Physiol..

[60] Behling-Kelly E. (2014). Serum Lipoprotein Changes in Dogs with Renal Disease. J. Vet. Intern. Med..

[61] Vaziri N.D., Norris K. (2011). Lipid disorders and their relevance to outcomes in chronic kidney disease. Blood Purif..

[62] Leal-Pinto E., Park H., King F., MacLeod M., Pitts R. (1973). Metabolism of lactate by the intact functioning kidney of the dog. Am. J. Physiol..

[63] Bellomo R., Kellum J.A., Pinsky M.R. (1996). Transvisceral lactate fluxes during early endotoxemia. Chest.

[64] Bartlett S., Espinal J., Janssens P., Ross B.D. (1984). The influence of renal function on lactate and glucose metabolism. Biochem. J..

[65] Kopple J.D., Swendseid M.E. (1975). Protein and amino acid metabolism in uremic patients undergoing maintenance hemodialysis. Kidney Int. Suppl..

[66] May R.C., Hara Y., Kelly R.A., Block K.P., Buse M.G., Mitch W.E. (1987). Branched-chain amino acid metabolism in rat muscle: Abnormal regulation in acidosis. Am. J. Physiol. Endocrinol. Metab..

[67] Busque S.M., Wagner C.A. (2009). Potassium restriction, high protein intake, and metabolic acidosis increase expression of the glutamine transporter SNAT3 (Slc38a3) in mouse kidney. Am. J. Physiol. Ren. Physiol..

[68] IRIS Staging of CKD. http://www.iris-kidney.com/guidelines/.

[69] FEDIAF—European Pet Food Industry Federation (2019). Nutritional Guidelines for Complete and Complementary Pet Food for Cats and Dogs.

[70] Beckonert O., Keun H.C., Ebbels T.M.D., Bundy J., Holmes E., Lindon J.C., Nicholson J.K. (2007). Metabolic profiling, metabolomic and metabonomic procedures for NMR spectroscopy of urine, plasma, serum and tissue extracts. Nat. Protoc..

