# Supplementary material for: Healthy and Chronic Kidney Disease (CKD) Dogs Have Differences in Serum Metabolomics and Renal Diet May Have Slowed Disease Progression
Source: Metabolites. 2021 Nov 16;11(11):782. doi: 10.3390/metabo11110782 (PMC8623449; doi:10.3390/metabo11110782)
Supplement: Supplementary file 1 [file metabolites-11-00782-s001.zip › metabolites-1434516-supplementary.pdf]

molecules

Article

Healthy and chronic kidney disease (CKD) dogs have differences in serum metabolomics and renal diet may have slowed disease progression

Marcio Antonio Brunetto [1,2], Bruna Ruberti [3], Doris Pereira Halfen [2], Douglas Segalla Caragelasco [3], Thiago Henrique Annibale Vendramini [1], Vivian Pedrinelli [2], Henrique Tobaro Macedo [1], Juliana Toloi Jeremias [4], Cristiana Fonseca Ferreira Pontieri [4], Fernanda Maria Marins Ocampos [5], Luiz Alberto Colnago[5], Marcia Mery Kogika[3,*]

[1] Pet Nutrology Research Center, Nutrition and Production Department, School of Veterinary Medicine and Animal Science, University of São Paulo, Jardim Elite, Pirassununga 13635-900, Brazil; mabrunetto@usp.br (M.A.B.); thiago.vendramini@usp.br (T.H.A.V.); henrique.tobaro@hotmail.com (H.T.M.)
[2] Veterinary Nutrology Service, Veterinary Teaching Hospital, School of Veterinary Medicine and Animal Science, University of São Paulo, Cidade Universitária, São Paulo 05508-270, Brazil; dorisph2@yahoo.com.br (D.P.H.); vivian.pedrinelli@usp.br (V.P.)
[3] Small Animal Internal Medicine Service, Veterinary Teaching Hospital, School of Veterinary Medicine and Animal Science, University of São Paulo, Cidade Universitária, São Paulo 05508-270, Brazil; brunaruberti@usp.br (B.R.); mv.douglas@yahoo.com.br (D.S.C.)
[4] Nutrition Development Center, Grandfood Industria e Comercio LTDA (Premier Pet®), Dourado, São Paulo, Brazil; jjeremias@premierpet.com.br (J.T.J.); cristiana@premierpet.com.br (C.F.F.P.)
[5] Embrapa Instrumentação, R. 15 de Novembro, 1452, Centro, São Carlos, SP 13560-970, Brazil; fmmocampos@gmail.com (F.M.M.O.); luiz.colnago@embrapa.br (L.A.C.)
* Correspondence: mabrunetto@usp.br (M.A.B.)

Supplementary information

Figures

Figure S1. *Comparison between 1D NOESY-presat and 1D CPMG ^{1}H NMR spectra (600 MHz, 300 K) of the QC sample, indicating the chemical shifts of the main detected metabolites of dog's serum, demonstrating the effect of the T2 filter.* *2*
Figure S2. *Periods, animals and experimental procedures that were performed in the study.* *2*
Figure S3. *Expansion (6.0 - --.2 ppm) of HSQC correlation map (^{1}H and ^{13}C at 600.13 and 150.90 MHz, respectively, 300 K) of the QC sample from dogs' serum* .... ***Erro! Indicador não definido.***
Figure S4. *Expansion (6.0 - --.2 ppm) of HMBC correlation map (^{1}H and ^{13}C at 600.13 and 150.90 MHz, respectively, 300 K) of the QC sample from dogs' serum.* .... ***Erro! Indicador não definido.***
Figure S5. *Expansion (6.0 - --.2 ppm) of COSY correlation map (^{1}H at 600.13 MHz, 300 K) of the QC sample from dogs' serum.* .... ***Erro! Indicador não definido.***

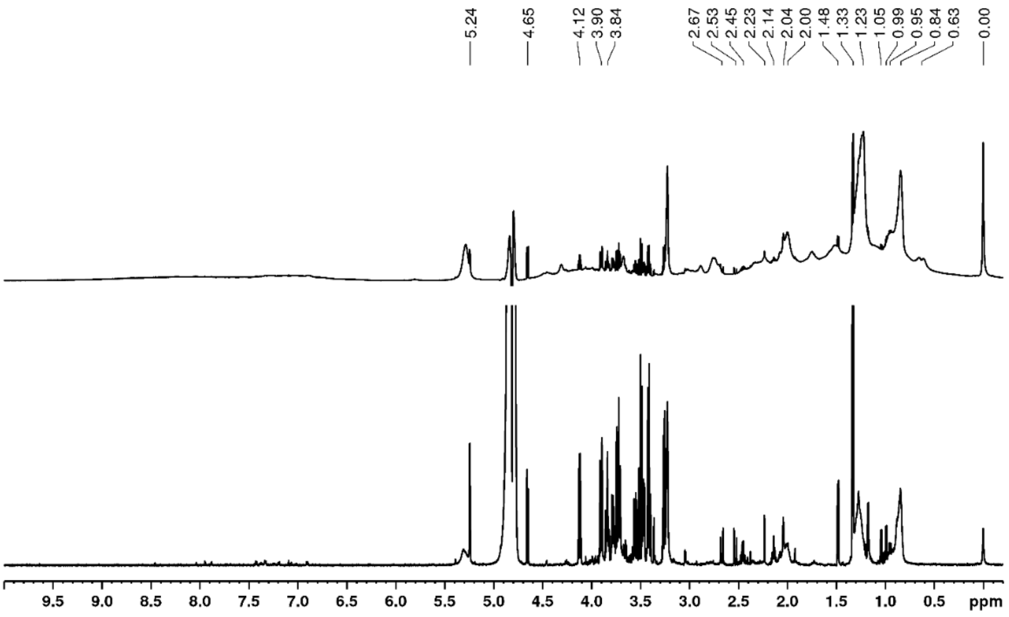

Figure S1. Comparison between 1D NOESY-presat and 1D CPMG ^{1}H NMR spectra (600 MHz, 300 K) of the QC sample, indicating the chemical shifts of the main detected metabolites of dog's serum, demonstrating the effect of the T2 filter.

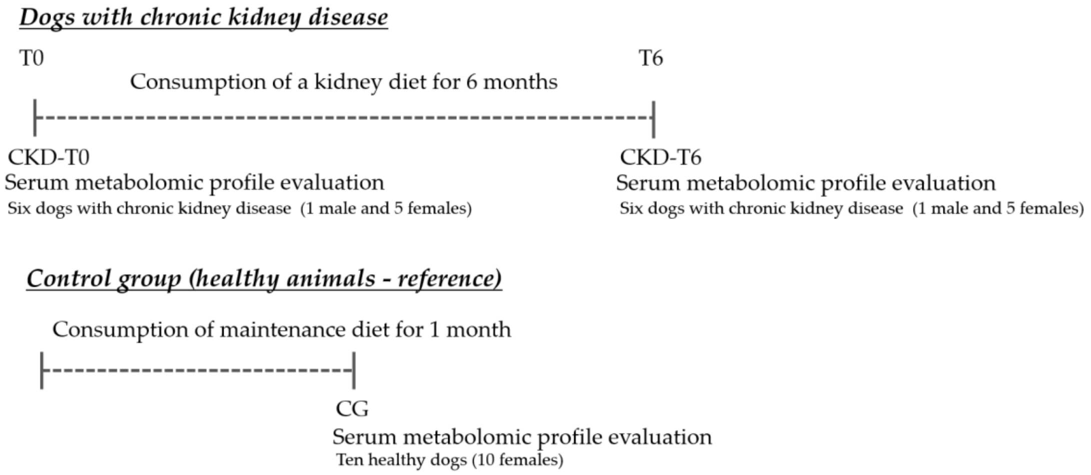

Figure S2. Periods, animals and experimental procedures that were performed in the study.

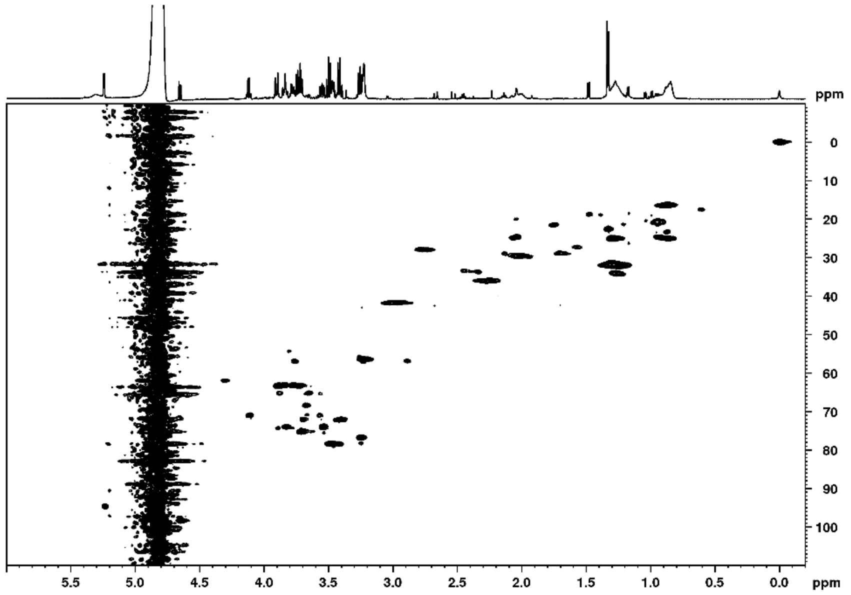

Figure S3. Expansion (6.0 - --.2 ppm) of HSQC correlation map (^{1}H and ^{13}C at 600.13 and 150.90 MHz, respectively, 300 K) of the QC sample from dogs' serum

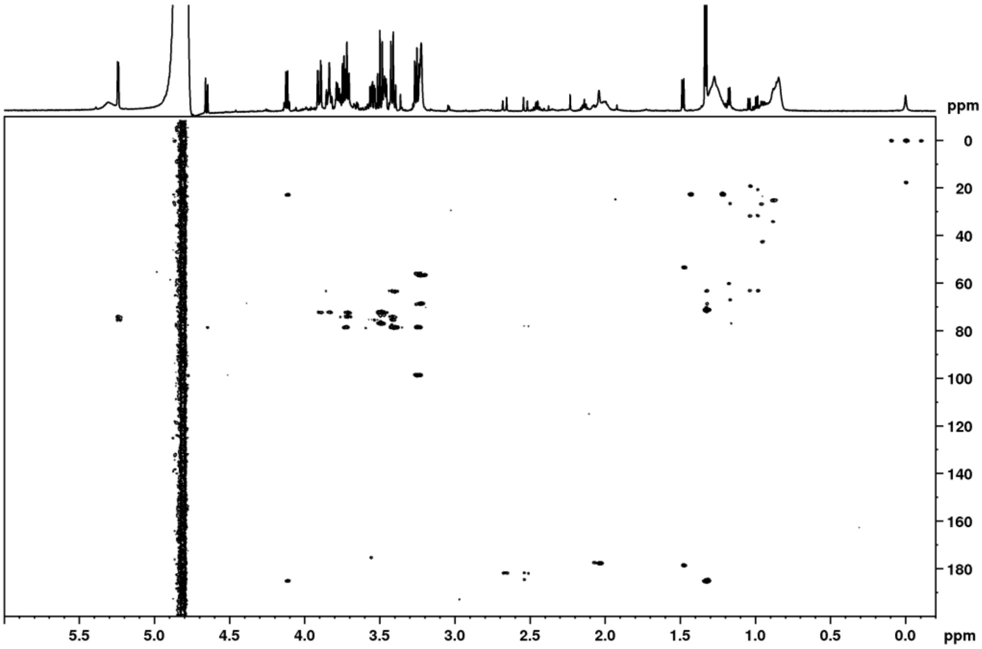

Figure S4. Expansion (6.0 - --.2 ppm) of HMBC correlation map (^{1}H and ^{13}C at 600.13 and 150.90 MHz, respectively, 300 K) of the QC sample from dogs' serum.

molecules

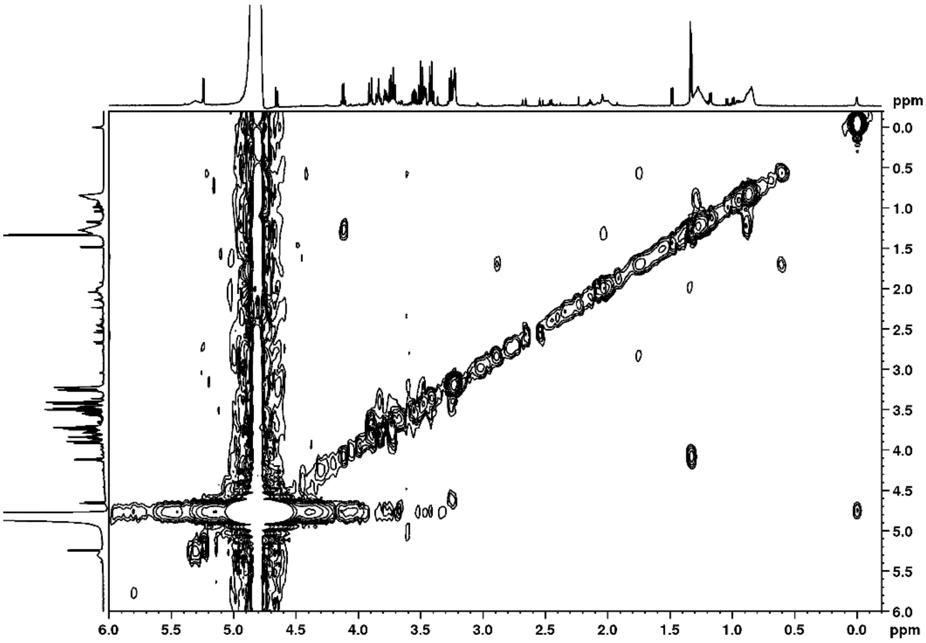

Figure S5. Expansion (6.0 - --.2 ppm) of COSY correlation map (^{1}H at 600.13 MHz, 300 K) of the QC sample from dogs' serum.
